# Feasibility of an innovative electronic mobile system to assist health workers to collect accurate, complete and timely data in a malaria control programme in a remote setting in Kenya

**DOI:** 10.1186/s12936-015-0965-z

**Published:** 2015-11-04

**Authors:** David O. Soti, Stephen N. Kinoti, Ahmeddin H. Omar, John Logedi, Teresa K. Mwendwa, Zahra Hirji, Santiago Ferro

**Affiliations:** Division of Health Informatics and Monitoring and Evaluation, Ministry of Health, PO Box 30016-00100, Nairobi, Kenya; Fio Corporation, Toronto, Canada; Department of Physiology, University of Nairobi, P O Box 52379-00200, Nairobi, Kenya

**Keywords:** Feasibility, Quality data, Health workers, Remote setting, mHealth

## Abstract

**Background:**

The cornerstone of decision making aimed at improving health services is accurate and timely health information. The Ministry of Public Health and Sanitation in Kenya decided to pilot feasibility of Fionet, an innovation that integrates diagnostics, data capture and cloud services, in its malaria control programme to demonstrate usability and feasibility by primary level workers in a remote setting in Kenya.

**Methods:**

Eleven sites comprising one sub-district hospital, ten health centres and dispensaries were selected in three districts of Kisumu County to participate. Two health workers per site were selected, trained over a two-day period in the use of the Deki Reader™ to undertake rapid diagnostic testing (RDT) for malaria and data capture of patients’ records. Health managers in the three districts were trained in the use of Fionet™ portal (web portal to cloud based information) to access the data uploaded by the Deki Readers. Field Support was provided by the Fio Corporation representative in Kenya.

**Results:**

A total of 5812 malaria RDTs were run and uploaded to the cloud database during this implementation research study. Uploaded data were automatically aggregated into predetermined reports for use by service managers and supervisors. The Deki Reader enhanced the performance of the health workers by not only guiding them through processing of a malaria RDT test, but also by doing the automated analysis of the RDT, capturing the image, determining whether the RDT was processed according to guidelines, and capturing full patient data for each patient encounter. Supervisors were able to perform remote Quality assurance/Quality control (QA/QC) activities almost in real time.

**Conclusion:**

Quality, complete and timely data collection by health workers in a remote setting in Kenya is feasible. This paperless innovation brought unprecedented quality control and quality assurance in diagnosis, care and data capture, all in the hands of the health worker at point of care in an integrated way.

**Electronic supplementary material:**

The online version of this article (doi:10.1186/s12936-015-0965-z) contains supplementary material, which is available to authorized users.

## Background

Health information infrastructure in developing countries continues to be one of the weakest components among the six building blocks considered by the World Health Organization to be necessary in order to achieve strengthening of health systems [1]. Fortunately most health leaders agree that accurate and timely health information is the cornerstone for decision-making, serving the key functions: data generation, compilation, analysis and synthesis, communication, and use [2].

A well-functioning and efficient information system is able to track the performance of health systems in general. As noted by Mutale et al. [3], weak health information systems (HIS) are a critical challenge to reaching the health-related Millennium Development Goals because health system performance cannot be adequately assessed or monitored where HIS data are incomplete, inaccurate or untimely. Making data more complete, more accurate and timelier are imperatives to strengthen health systems as all the other building blocks depend on it. In order to achieve this, capacity of front-line workers at point of care in rural areas of developing countries must be strengthened. This must include provision of tools to assist in data capture and transmission.

Recent efforts towards strengthening information infrastructure have led to the development of the District Health Information System (DHIS). This is aimed at collection of health data and aggregation for analysis purposes to support decision-making. The latest version, DHIS 2, has been adopted by 30 countries in Africa, Asia, Latin America, and the South Pacific. Countries that have adopted DHIS 2 as their nationwide HIS software include Kenya, Tanzania, Uganda, Rwanda, Ghana, Liberia, and Bangladesh. Other mHealth or e-Health systems were reviewed in detail in the mHealth Compendium published by African Strategies for Health (ASH) Project in 2013 [4].

The quality of data in the DHIS, as well as in other similar systems, is as good as the data that feeds into it from facilities below District. Data capture at point-of-care (POC) in the community, rural dispensaries, health centres, and sub-district hospitals is mostly paper-based and depends on few, often overworked, health workers. Such workers have very little supervision and have no tools to digitally collect data and transmit it to their managers in a timely manner. This dilemma has been recognized and is being addressed by review of various technologies [4] that are being introduced, as discussed by African Strategies for Health (ASH) in the mHealth Compendium 2013.

Quality and complete data needs to be transmitted, aggregated, analysed, and transformed into information that can be communicated and used for decision making. Digitally generated data makes it easier to transmit, aggregate and analyse. Fionet (Fio Corporation, Toronto, Canada) is an integrated digital health collection and reporting system using mobile devices to help health workers deliver quality healthcare services, automatically capturing and disseminating primary data to a central web portal. Devices currently compatible with Fionet include: android smartphones, tablets and the Deki Reader™. The Deki Reader is a mobile, in vitro diagnostic device that interprets commercially available rapid diagnostic tests (RDTs) for infectious diseases such as malaria, HIV, syphilis, hepatitis B, and dengue, among others. Deki mobile software applications integrate clinical workflow guidance and digital data capture at POC, then transmit geo-tagged records of patient encounters to a secure cloud database. The Fionet portal allows health programme stakeholders to remotely monitor diagnostic performance, adherence to case management protocols, healthcare workers’ activity levels, and other health performance indicators. The performance of the Deki Reader as an in vitro diagnostic tool has been validated [5, 6] in field conditions.

Many health information technologies (HIT), despite being promising, have failed when implementation attempts were made in low-resource settings. Therefore, it is warranted to conduct implementation research studies to evaluate the feasibility and usability of new HIT in developing countries [7–9].

This paper presents results of implementation research to evaluate the feasibility and usability of Fionet system new technology by healthcare workers in remote locations in rural Kenya and to collect feedback on the system from intended Deki users using Fionet.

## Methods

### Selection of field sites and Deki Reader users

**Fig. 1:**
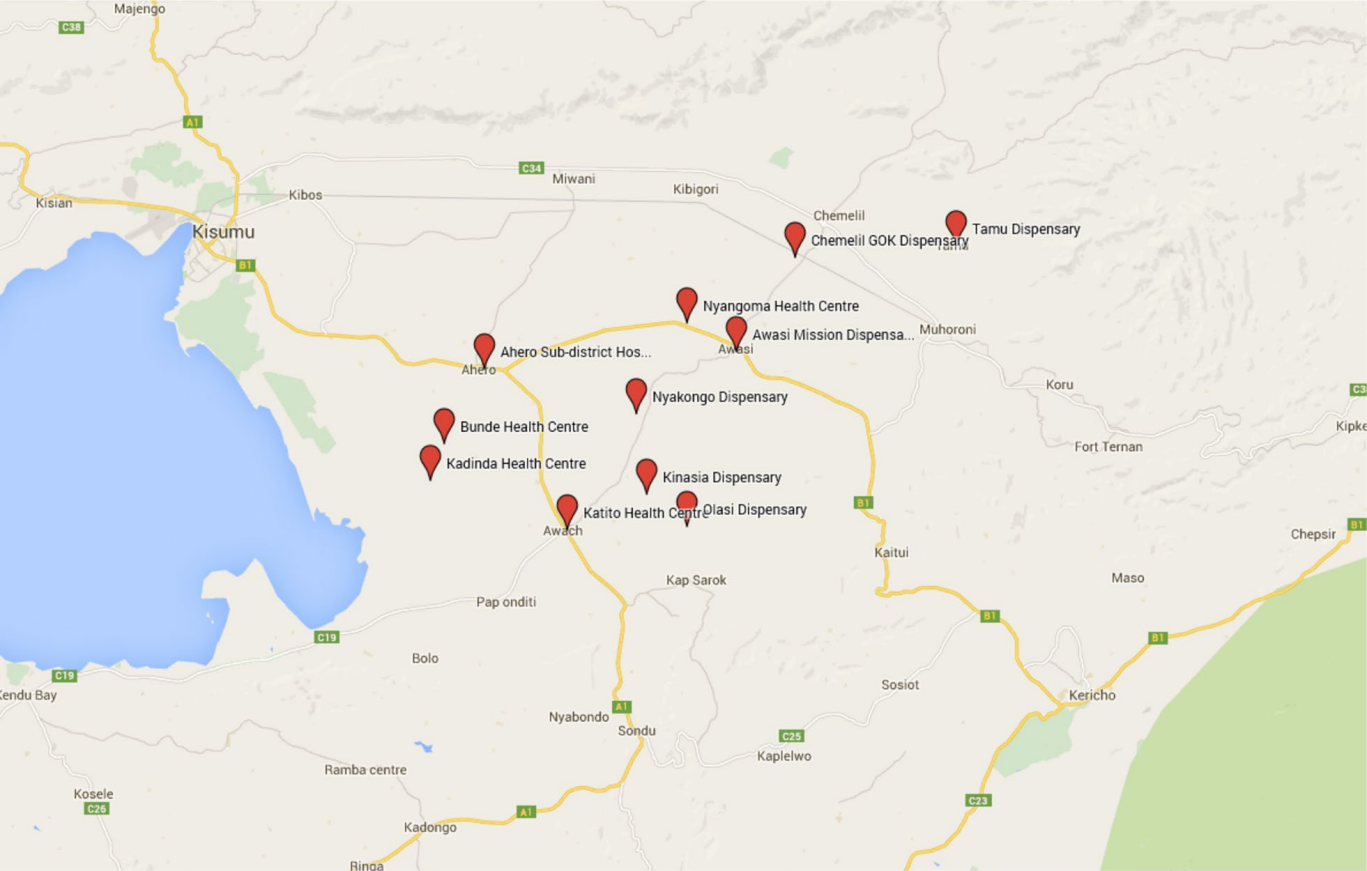
Facility locations

With the objective to demonstrate feasibility of quality and timely data collection by health workers in a remote setting in Kenya, 11 sites comprising ten health centres and one sub-district hospital were selected in Kisumu County (Fig. 1). From each of these sites two health workers were selected by the Malaria Control Unit, Ministry of Health, Kenya. Deki Reader units were allocated in health centres, clinics and/or community dispensaries that the control unit identified as programme participants. Because the aim of the programme is to improve diagnosis and treatment of malaria, the Deki Reader users selected were those that were already familiar with processing of malaria RDTs.

### Data capture

The data-capture forms to be used were defined by the investigators, reviewed and approved by the malaria control unit and subsequently programmed into the Deki Readers. Fio Corporation established a secure Portal to which the data from the Deki Readers were uploaded for easy access by programme managers. Malaria Control Unit (MCU) also designed preconfigured aggregation reports and data mining views that were developed by Fio Corporation and installed in the MCU Portal prior to the start of the feasibility implementation study.

### Field support to Deki users and programme managers

Field support was provided by an IT specialist of Viva Afya, Fio’s local partner, and by MCU’s IT specialist. Software and hardware-related assistance was provided remotely by Fio’s software engineers in Toronto, Canada.

### Timeline

The feasibility evaluation was undertaken over an eight-week period in May and June 2013.

### Feasibility and usability measures

Malaria Control Unit and the investigators agreed on the following feasibility and usability measures and targets: timeliness of data capture, transmission and aggregation of more than 95 % in the first week; completeness of individual datasets of greater than 96 %; accuracy of individual records greater than 96 %; and, proportion of time information services available through Fionet portal (portal to cloud services). Because Fionet is automated, the results of these measures were available from the cloud service database throughout the study period. These target thresholds were considered to be stringent criteria to be able to make a final assessment of the feasibility and usability of the Fionet system.

At the end of the pilot, all 23 users completed a qualitative usability survey on their experience with the Deki Reader. Users were asked to provide feedback regarding three main features of the reader: the user interface, RDT workflow, and test results. The Additional file 1: Appendix provides the survey questions for each of these features. Each response was ranked on a qualitative scale consistent of four options: ‘Very good’, ‘Somewhat’, ‘A little’, and ‘Not at all’.

### Training for the pilot evaluation

Fionet training was conducted over a two-day period. The training was divided into three sessions: classroom training, clinical practice and Fionet portal training. Trainees included 22 practitioners and 19 administrators. All trainees attended the classroom and clinical training; in addition, programme administrators attended Fionet portal web portal training.

#### **Classroom training**

Classroom training session familiarized trainees with the Deki Reader and its basic operations. Trainees learned how to log patient and test information using electronic survey forms and RDTs. Additionally, trainees were provided with a refresher on RDT processing and the Deki Reader’s RDT processing quality control features. While the Deki Readers initially detected a lot of improperly run RDTs due to too much blood being added, as training progressed, particularly in the clinical practice session, the quality of RDT processing increased and errors were reduced.

#### **Clinical practical training**

Clinical practice session divided trainees into two teams and consisted of a full-day clinical practice with the Deki Readers at Ahero Sub-District Hospital and Nyangoma Health Centre. The trainees processed RDTs on real patients and became familiar with the clinical workflow. Additionally, trainees used the Deki Readers to process multiple patients simultaneously in order to accelerate their performance. By the end of the session, all the users were processing multiple patients easily with very few quality errors reported by the Deki Reader.

#### **Fionet portal training**

Fionet portal training session familiarized administrators with how Fionet portal stores and reports patient records. Fionet portal trainees learned how to monitor the daily, weekly and monthly test results, run reports, and query and filter data. In addition, trainees were taught how to manage quality issues, including how to identify and intervene when users experienced challenges in processing RDTs.

### Data analysis

Descriptive statistics were used to summarize the data collected. Information was analysed both collectively and by participating study site. General demographic data were summarized using common statistical tests, such as average and mean. Deki users were asked to rank how usable the Deki Readers were for three key aspects: user interface, workflow and test results. For each of the items evaluated, users were asked to use a predefined scale designed to capture the level of agreement of the user with each of the presented statements (see Additional file 1). The ‘User Interface’ domain consisted of 14 sentences, the ‘Workflow’ domain had seven questions and the ‘Test Results’ three. For each sentence presented the average response was calculated by assigning a numeral value to the qualitative options as follows: ‘Very good’ 4 points, ‘Somewhat’ 3 points, ‘A little’ 2 points, and ‘Not at all’ 1 point.

## Results

### Data capture, transmission and aggregation

A total of 5812 malaria RDTs were run and uploaded to the cloud database during the programme. Table 1 gives the demographics of the patient population seen and tested for malaria during the evaluation.

**Table 1:** Summary of demographics of patient population

Age (years)	# Tests					
	Females		Males		Total	
	n	%	n	%	n	%
Under 5	767	47.6	843	52.4	1610	27.7
5 to 15	1020	52.8	912	47.2	1932	33.2
16 to 45	1189	68.6	543	31.4	1732	29.8
46+	351	65.2	187	34.8	538	9.3
Total	3327	57.2	2485	42.8	5812	100

**Fig. 2:**
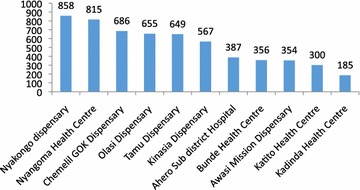
Records uploaded by facility

The average number of records uploaded into the Fionet cloud per facility was 528. Uploaded data were automatically aggregated into predetermined reports for use by service managers and supervisors. The total number of uploads by facility represent the numbers of patients seen in Fig. 2.

Since all tests processed by the Deki led to capture of the data and transmission in real time, this reflects the capability of the Deki Reader to assure complete data capture even from remote rural areas.

### Timeliness of data capture and transmission

**Fig. 3:**
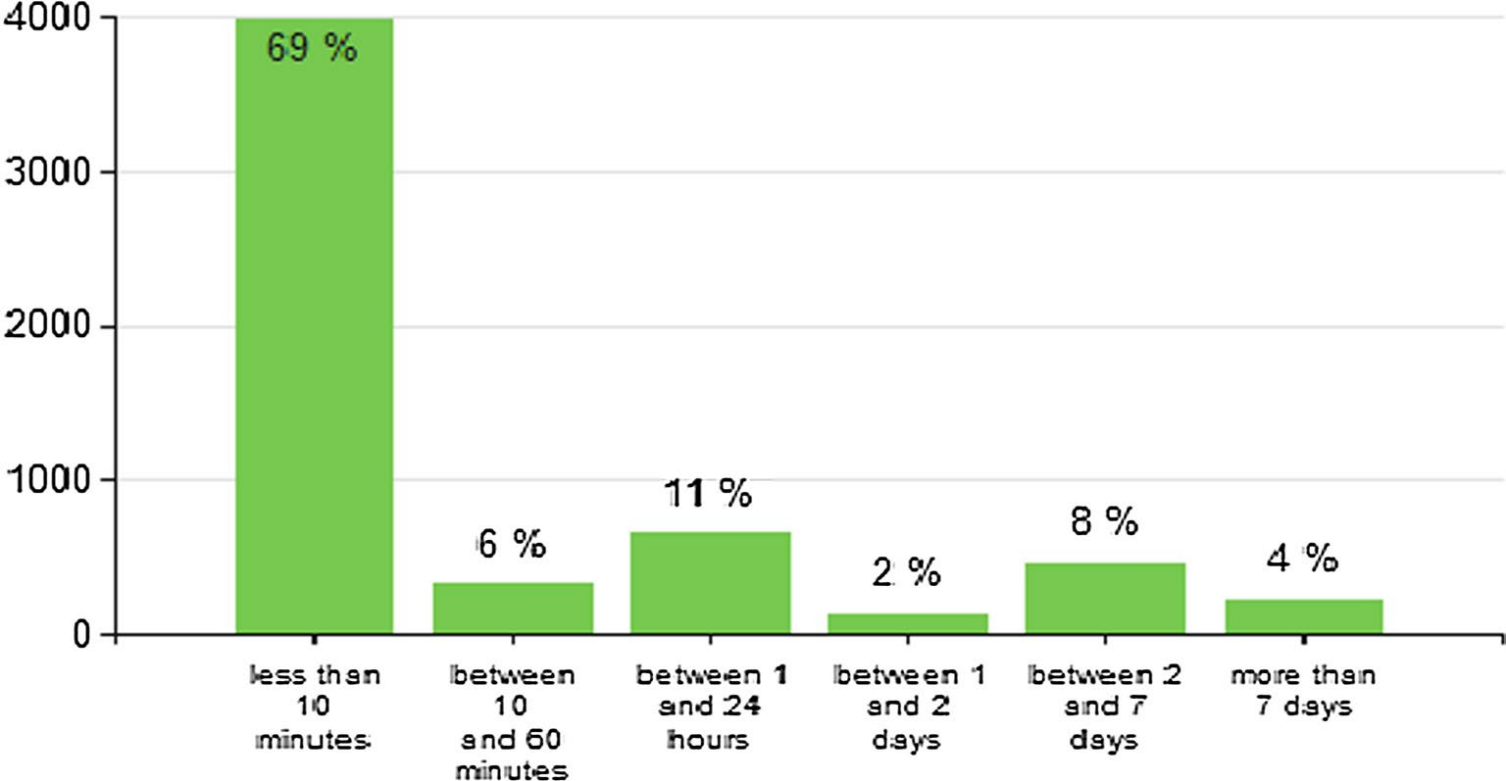
Record upload speed

Upload times of completed patient records to the cloud database were monitored during the pilot. As shown in Fig. 3, 86 % of completed records were received within 24 h and 96 % of completed records were received within 1 week after completion of the case. In addition, users did not report any difficulties in relation to recharging the batteries in the unit. The high number of records uploaded daily by each of the facilities demonstrates no major issues related to mobile network connectivity nor to recharging batteries of the mobile units.

### Overall performance in respect to feasibility measures

At the outset of the programme, seven performance measures were agreed upon by investigators. These included timeliness of data aggregation; completeness of individual datasets; accuracy of individual records; data exportability; proportion of Deki unit service requests responded to within 72 h; proportion of Deki unit support queries responded to within 72 h of initiation; and proportion of time information services were available through Fionet portal. Table 2 provides the predetermined targets and actual performance achieved during the programme. As shown, all the targets were met and exceeded.

**Table 2:** Summary of feasibility of quality and timely data collection measures

	Measure	Target	Achieved
1.	Timeliness of data aggregation as measured by average time difference between completion of dataset at point of care and its arrival in database barring unforeseen mobile network interruptions or connection problems	>95 % within 1 week	96 % within 1 week
2.	Completeness of individual datasets as measured by average number of data fields containing response (includes Ministry of Health official malaria case report forms, RDT results and image when applicable, user and facility information, and meta information such as date, time. Note: GPS location is excluded due to dependencies on weather patterns and building location)	>96 %	97.4 %
3.	Accuracy of individual records as measured by percentage of data fields containing responses within pre-defined allowable ranges (includes mandatory forms, RDT results and image, patient demographic information, user and facility information, and meta information such as date, time)	>96 %	>99.9 %
4.	Data can be exported in XML, XLS, CSV formats	Yes/No	Yes
5.	Proportion of time information services available through Fionet portal	95 %	>99 %
6.	Proportion of Deki unit support queries responded to within 72 h of initiation	>95 %	100 %
7.	Proportion of Deki unit service requests responded to within 72 h of initiation	>95 %	100 %

### Usability of Deki devices

**Fig. 4:**
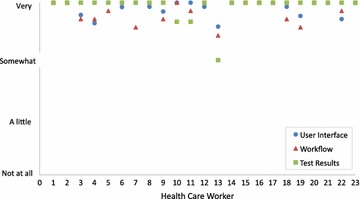
Usability Survey Results by Health Care Worker

User responses in relation to user interface, workflow and test results are shown in Fig. 4. On average, Deki users found the devices easy to use. They did not report issues related to difficulties in the processing of RDTs through the Deki. They reported that the use the device not only did not interfere with their routine activities, but it helped them to perform their job more efficiently. The number of patients seen at each of the facilities was more or less the same as the number usual attending. Users did not report any difficulties with recharging the batteries in the unit. The high number of records uploaded daily by each of the facilities demonstrates no major issues related to mobile network connectivity, nor to recharging batteries of the mobile units.

## Discussion

The main objective of the present implementation study was to test an innovative system, based on modern communications technology, to assist the Malaria Control Unit activities under real conditions in the public health sector in Kisumu County, rural Kenya. The conditions of the location selected for the implementation study are comparable to conditions in many other malaria-endemic countries, and are characterized by being low-income communities, economically active in small agricultural activities, with scarce means of transportation, and very limited housing conditions, in many cases without basic services (portable water, electricity, sewage system, etc.) but with reasonably good mobile phone network coverage. The results obtained in the present experience can be generalized to other locations in malaria-endemic areas of Africa.

The sound decision-making at all levels of the health system requires reliable, timely and quality information that are presented by location, sex, age, socio-economic status, and other characteristics. At policy level, decisions informed by evidence contribute to more efficient resource allocation and, at delivery level, information about quality and effectiveness of services can contribute to better outcomes. Information systems, particularly at POC, need to be simple and sustainable and not overburden health delivery staff or be too costly to run. Considering the results observed in the present implementation, the Fionet system was demonstrated to be a useful tool to accomplish all of the above mentioned tasks.

In traditional, paper-based, data recording systems, still present in most rural settings in developing countries of Africa, because of the many forms and registers to be filled from as many data sources, data are often inaccurate, illegible and incomplete. It has been very difficult to know whether what is reported is really what the health worker does. Use of Fionet system in the current deployment eliminated the need for paper records by having all the required indicators preprogrammed into electronic forms and pushed into the Deki devices. The health worker used menus that ensured standard protocols are followed and no missed fields in data capture. This in turn helped to obtain completeness of data, including capture of invalid processes of care or testing, which provided an opportunity for remote quality control, support and corrective measures to be taken.

The Deki Reader enhanced the performance of the health workers by not only guiding them through processing of a test, but also doing an automated digital analysis of the RDT, capturing the image, performing on-site quality control of RDT process, giving immediate feedback to the health worker and capturing full patient data for each patient encounter. The Deki Readers transmitted all this data to the supervisors who accessed it through the Fionet portal. Upon noticing care management-related problems, the supervisors gave prompt feedback to the health workers. The supervisors periodically, through log-into the Fionet portal, were able to readily access data collected by each healthcare facility, prepare a report and share it with each of the participating centres. Data captured at point of care was automatically available for review on the Fionet portal through individual records and aggregated reports. These reports could be shared with personnel from all health facilities participating in the implementation study, as well as with higher levels of the Malaria Control Unit management team, Minister of Health officers, etc.

In the implementation effort reported here, Fionet showed many attractive features and was highly regarded by healthcare workers in the field as a useful tool to increase their efficiency, and by supervisors as a great resource for quality control of both diagnostics and patient case management. The cost advantages of the implementation of a system like this can be anticipated since it strengthens the overall healthcare system. A recent cost-benefit investigation considering improvements in diagnostic quality and case management showed that deploying Fionet to strengthen malaria RDT-based case management, results in cost savings per Disability Adjusted Life Years (DALY) averted, in other words, reduced disease burden is observed while spending less per patient [10]. Additional cost savings were found when including improvements in data collection and appropriate treatment. Furthermore, after a successful pilot conducted recently in Meru County, Kenya, the local government is funding a countywide implementation of Fionet covering all level 2 and level 3 health facilities. This endorsement points to the affordability, scalability and sustainability of Fionet (William Muraah, pers. comm.).

Staff need frequent feedback on how routine practices are being performed (mainly diagnostics and case management); but also on utility of data they collect to understand the importance of good quality data for improving health. Capacity building is required to ensure policymakers at all levels have the ability to use and interpret health data. It is also important that health system staff understand the significance of local data for local programme management, and that their needs for strengthened capacity for critical health statistical analysis are met. Local use of data collected at lower levels of the health system is a key step for improving overall data quality. This paperless, mHealth innovation brought about unprecedented quality control and quality assurance in diagnosis of malaria, patient care and data capture, all in the hands of the health worker at point of care in an integrated way. This finding is in agreement with what has previously been demonstrated [11]. The malaria programme in the Kisumu area successfully tested the Fionet system for its ability to enable remote quality assurance and control in areas where otherwise it would have been more costly and cumbersome to perform following current recommendations [12, 13].

The outcome measurements of the study met the expectations in terms of accuracy, completeness, timeliness, transmission, and use of real-time data for feedback and support of health workers in a remote setting in Kenya, as shown in Table 2. Implementation of a system such as Fionet on a larger scale, such as a whole district level, is therefore warranted and being implemented in Kenya. This is a reliable indicator of the feasibility of the system in the hands of intended users, and the ultimate test to determine scalability of the innovation. Because this tool is based on mobile technology, it is expected that its capability will expand as the mobile technology penetration increases in developing countries, especially in Africa [11].

## Conclusion

Innovative technologies can greatly improve the delivery of quality health care in remote locations of low- and middle-income countries. Quality, complete and timely data collection by health workers in a remote setting in Kenya is feasible. Such data can be transmitted, aggregated and used in real time to make service-related decisions and to remotely support healthcare workers to improve the services they provide.

## Additional file

10.1186/s12936-015-0965-z Usability survey.
