# Supplementary material for: Feasibility of an innovative electronic mobile system to assist health workers to collect accurate, complete and timely data in a malaria control programme in a remote setting in Kenya
Source: Malar J. 2015 Nov 4;14:430. doi: 10.1186/s12936-015-0965-z (PMC4632488; doi:10.1186/s12936-015-0965-z)
Supplement: Supplementary file 1 — 10.1186/s12936-015-0965-z Usability survey. [file 12936_2015_965_MOESM1_ESM.pdf]

Appendix 1

<u>Usability Survey</u>

<u>PART A</u>

Instructions: For each of the following statements, mark one box:

User Interface

	Very	Somewhat	A little	Not at all	Don't know
1. Did you find the User Manual easy to follow?					
2. Did you understand the warning and caution statements in the User Manual?					
3. Did you understand the biohazard precautions in the User Manual?					
4. Did you understand the Training Requirements section of the User Manual?					
5. Was the stylus helpful when using the reader?					
6. Did you find it easy to type on the reader?					
7. Did you understand the icons on the screen?					
8. Was it easy to insert and remove RDTs from the reader?					
9. Were the instructions for charging the reader easy to understand?					

10. Did you find it easy to log ***into*** the reader?					
11. Did you find it easy to log ***out*** of the reader?					
12. Did you find it easy to perform a Functional Check?					
13. Did you find the reader easy to clean?					
14. Was the reader easy to carry?					

Workflow

	**Very**	**Somewhat**	**A little**	**Not at all**	**Don't know**
1. Were the onscreen prompts clear and easy to follow?					
2. Did the reader guide your RDT workflow?					
3. Did the reader help in patient management and capturing patient information?					
4. Did the reader help you to keep track of multiple patients and/or tests?					
5. Was the timer on the reader helpful?					
6. Did the timer beep help you analyse RDTs on time?					

7. Did you find it easy to clear a patient from the Patient Manager list (i.e., complete a patient test)?					

Test Results

	**Very**	**Somewhat**	**A little**	**Not at all**	**Don't know**
1. Were the test results easy to read?					
2. Did you understand the test results?					
3. Did you find the Deki Reader useful?					

PART B

Instructions: Please answer the following questions about the Deki Reader features.

1. What features if the reader did you like the best?
   …………………………………………………………….
   …………………………………………………………….

2. If you could change anything about the reader, what would it be?
   ……………………………………………………..
   …………………………………………………………….

3. Please provide any additional comments about the reader.
   ………………………………………………………
   …………………………………………………………….
